# Genetic characterization of an isolate of HIV type 1 AG recombinant form circulating in Siberia, Russia

**DOI:** 10.1007/s00705-012-1442-4

**Published:** 2012-08-19

**Authors:** P. B. Baryshev, V. V. Bogachev, N. M. Gashnikova

**Affiliations:** State Research Center of Virology and Biotechnology VECTOR, Novosibirsk, Russia

## Abstract

Before 2008, HIV-1 subtype A was the predominant genetic variant in the Novosibirsk oblast of Russia as well as in most parts of this country. However, a rapid spread of the recombinant HIV-1 02_AG form has been reported in Novosibirsk since 2009. We have analyzed the genome of the 10.RU.6637 isolate, a HIV-1 02_AG recombinant form, which represents a monophyletic cluster of the HIV-1 variants widespread in this region. Phylogenetic analysis has shown that the Siberian 10.RU.6637 isolate displays the highest sequence identity to the HIV-1 subtype AG forms circulating in Uzbekistan. However, recombination analysis of 10.RU.6637 has demonstrated that this isolate is a recombinant form between HIV-1 subtype A and CRF02_AG, differing in its genetic structure from both the CRF02_AG reference sequences and the Central Asian variants of HIV-1 02_AG.

## Introduction

Recombination is one of the driving forces in HIV evolution. Recombination increases genetic diversity of the virus and leads to emergence of new epidemically important HIV-1 forms [1–4]. At least 20 % of the HIV-1 isolates circulating in the world are recombinants of different virus subtypes [5, 6]. The HIV-1 recombinant forms fall into two categories, namely, circulating recombinant forms (CRFs) and unique recombinant forms (URFs), i.e., the viruses that are widespread in a certain area and have a stable genetic structure in the population and the viruses with limited ability to spread, respectively [7]. Currently, more than 45 CRFs and 100 URFs of HIV-1 are known, and their number is continuously growing (http://www.hiv.lanl.gov).

CRF02_AG is the most abundant among the HIV-1 recombinant forms [8, 9]. First identified in Nigeria in 1994 [10], this genetic variant has not only become predominant in Western Africa, but it is also widespread in many countries on other continents [7, 11]. Particularly, CRF02_AG variants in several Central Asian countries rank second to HIV-1 subtype A in the number of people infected [12, 13].

According to some researchers, HIV-1 subtype A has been prevalent in majority of Russian regions since the mid-1990s [14, 15]. The same scenario was observed in the Novosibirsk oblast until recently. However, the emergence of the HIV-1 02_AG recombinant form was recorded in 2006, and this genetic variant has become most abundant among the HIV-1 subtypes. Phylogenetic analysis of the studied HIV-1 02_AG variants has shown that the recombinant forms comprise at least three groups of viruses: (1) rare HIV isolates that are genetically related to the HIV-1 02_AG forms widespread in Cameroon, (2) HIV isolates with rather limited prevalence displaying similarity to the 02_AG variants circulating in Central Asia, and (3) actively spreading HIV-1 02_AG viruses that form a separate monophyletic cluster. The last group of viruses is the most numerous, accounting for more than 95 % of the HIV-1 02_AG recombinant isolates recovered in Siberia [16]. Earlier work on detection of HIV-1 02_AG in the Novosibirsk oblast included examination of a *pol* gene segment (with a length of 1300 bp) and a fragment of the V3 loop region of the HIV-1 *env* gene; however, this is insufficient for characterization of the new HIV-1 recombinant variant in Siberia. Hence, we report here the full genome sequence of the 10.RU.6637 isolate, which belongs to the group of the HIV-1 02_AG genetic variants most prevalent in Siberia. This isolate was recovered from an HIV-infected Novosibirsk citizen, its genetic properties were comprehensively analyzed, its phylogenetic relationships were determined, and recombination breakpoints were identified.

## Materials and methods

### RNA extraction

The 10.RU.6637 isolate was recovered in 2009 from a 28-year-old man who was infected in 2008 through a heterosexual contact. RNA was extracted from 500 μl of the plasma using ViroSeq reagents (Celera Diagnostics, Alameda, US).

### Reverse transcription, amplification, and sequencing

RNA reverse transcription with poly(dT) priming was performed using VIF-VPUoutR1 [17] in the *vpu* gene. The extracted RNA (3 μl) was reverse-transcribed in a total volume of 20 μl with 500 μM dNTPs, 2.5 mM primers, 10Ч RT buffer, 5 mM MgCl_2_, 10 mM DTT, 40 U of RNaseOUT, and 400 U of SuperScript III RNase H2 RT (Invitrogen, Carlsbad, USA).

Three regions of the viral genome were independently amplified from the cDNA using nested PCR to obtain a nearly full-length HIV-1 genome. The first amplicon started in the middle of the LTR (long terminal repeat) covering the *gag* gene and part of the *pol* gene (according to HXB2, positions 495–3338). The second spanned *pol* to *vpu* (HXB2, positions 2483–6231), and the third contained *env* and extended to the end of the LTR (HXB2, positions 5861–9632). The reaction mixture with a volume of 50 μl contained 1Ч PCR buffer, 350 μM dNTP mixture, 0.4 mM of each primer, and 5 U of Expand Long Template PCR enzyme mixture (Roche Diagnostics, Indianapolis, USA). Cycling conditions comprised the initial stage at 94 °C for 2 min; 10 cycles of 94 °C for 10 s, 60 °C for 30 s, and 68 °C for 3–4 min; 20 cycles of 94 °C for 10 s, 55 °C for 30 s, and 68 °C for 3–4 min; and a final stage at 68 °C for 10 min.

All amplicons were sequenced in an ABI 3130 automated sequencer (Applied Biosystems, Inc., Foster City, USA). DNA sequences were assembled using the Sequencher software (GeneCodes Inc., Ann Arbor, USA).

### Genetic analysis

The full genome sequences of 183 strains representing the main HIV-1 subtypes were downloaded from the Los Alamos database (http://www.hiv.lanl.gov sequence alignment page). All sequences were aligned using the Muscle software (http://www.drive5.com/muscle page). Alignment quality was manually checked in BioEdit [18] to ensure that the alignments did not contain obvious errors.

The jumping profile hidden Markov model (jpHMM) program [19, 20] was used to analyze the subtype assignment of all the retrieved sequences. In the jpHMM approach, each HIV-1 subtype is represented by a profile hidden Markov model. All profile models are connected by empirical probabilities, allowing for detection of possible recombinants and the related breakpoints by jumping from one profile to another.

The SimPlot v. 3.5.1 software [21] was initially used for bootscanning analysis of a query sequence against a set of other sequences. In the bootscanning plot, the phylogenetic relationships between the query sequence and the reference set was calculated using bootstrap resampling, and the bootstrap values were plotted along the genome. In this analysis, a window size of 300 nt and a step size of 20 nt were used.

Phylogenetic trees were constructed with the PhyML v. 3.0 program using the maximum-likelihood approach [22]. The statistical robustness of the maximum-likelihood topologies was assessed by bootstrapping with 100 replicates.

### Nucleotide sequence

The genomic sequence of HIV-1 10.RU.6637 isolate from Novosibirsk, Russia, was submitted to GenBank under accession number JN230353.

## Results

The genome of the HIV-1 10.RU.6637 isolate, recovered from a patient living in Novosibirsk, was sequenced. The sequenced genome has a length of 9187 bp. The studied genome sequence starts in the 5′LTR R region (HXB2, position 494) and terminates at the end of the 3′LTR R region (HXB2, position 9636).

**Fig. 1:**
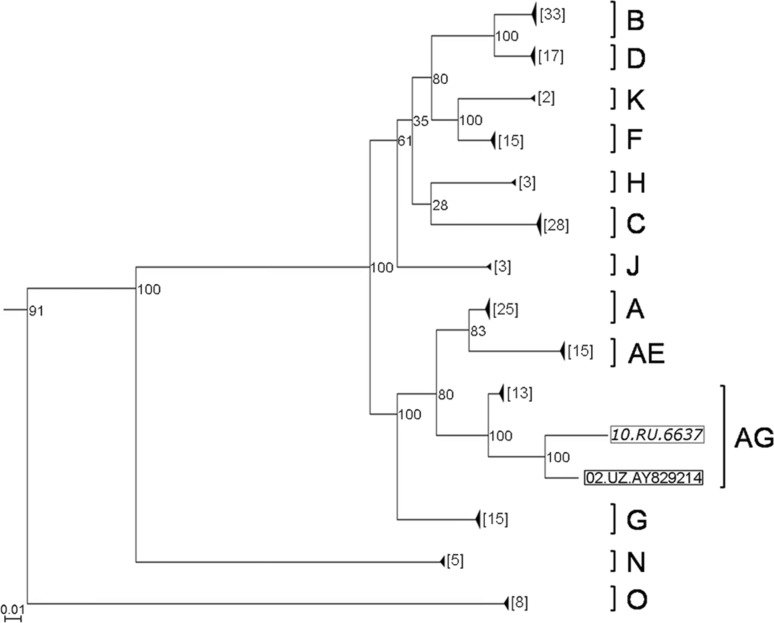
**Phylogenetic tree of HIV-1 full genome sequences**. The tree was constructed using the maximum-likelihood approach, using the genome sequences of the main HIV-1 subtypes from the Los Alamos database as well as CRF01_AE and CRF02_AG sequences. The monophyletic branches containing sequences of one subtype were collapsed to facilitate phylogenetic analysis. The number of genome sequences belonging to a given clade is shown in square brackets to the left. The studied HIV-1 genome (10.RU.6637 isolate) and the genome of HIV-1 from Uzbekistan (02.UZ.AY829214 isolate), which is nearest in the branch, are marked with square brackets

A sample of the full-genome sequences of the main HIV-1 subtypes was constructed for a comprehensive study of the HIV-1 10.RU.6637 genome. The constructed phylogenetic tree shows that the Uzbek variant of the HIV-1 AG recombinant form displays the highest sequence identity to the 10.RU.6637 isolate (Fig. 1). Bootstrap analysis confirmed that the topology where the Siberian sequence clusters in the same branch with the variant from Uzbekistan is statistically significant. Earlier, we determined HIV-1 *pol* gene sequences with a length of 1300 bp isolated from patients living in the Novosibirsk oblast. A considerable proportion of these samples showed a close similarity to the Central Asian HIV-1 variants on the phylogenetic tree but formed a separate monophyletic branch [16]. The sequence of the full genome of HIV-1 10.RU.6637 shows that it belongs to this branch.

**Fig. 2:**
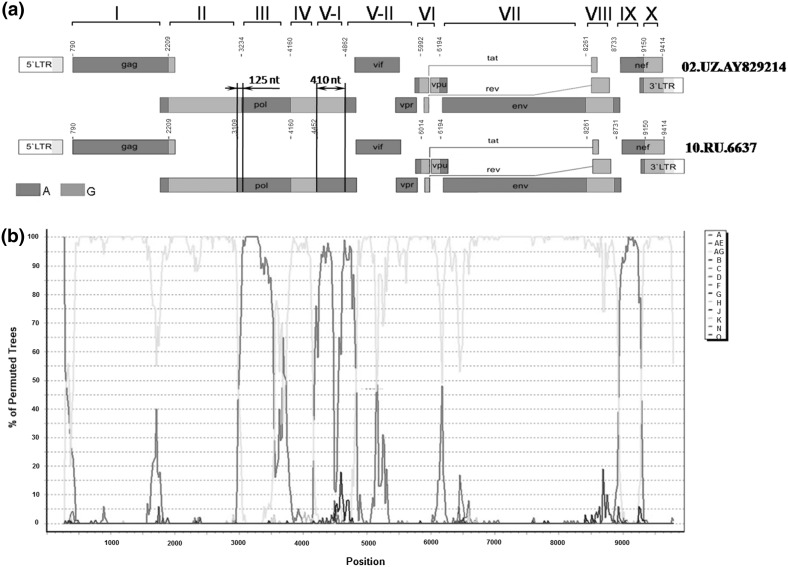
**Scheme of breakpoints in the 10.RU.6637 genome**. **a** Comparison of the breakpoints in 10.RU.6637 (bottom) and 02.UZ.AY829214 (top) genome sequences determined by jpHMM. The regions between the breakpoints are numbered from I to X. Region V is divided into two subregions, V-I and V-II. Arrows point to the differences in breakpoint positions of the compared HIV-1 genomes (regions II/III and IV/V-I). **b** SimPlot diagram. The sample of HIV-1 genome sequences used for analysis contained the sequences of different subtypes, including the circulating HIV-1 recombinant forms 02_AG and 01_AE

We hypothesized that an additional recombination event may have happened, giving rise to a new HIV-1 AG recombinant form with a genetic structure similar to the Central Asian HIV variants. To clarify whether we could speak about the emergence of a new HIV-1 AG recombinant form circulating in the Novosibirsk oblast, it was necessary to identify the locations of potential recombination breakpoints in the studied genome sequence. The jpHMM and SimPlot methods were used to identify such breakpoints. The constructed phylogenetic tree suggests that the HIV-1 02.UZ.AY829214 variant from Uzbekistan displays the highest similarity to our HIV-1 10.RU.6637 isolate; correspondingly, the breakpoint positions of these genome sequences were compared. The jpHMM results are shown in Table 1 and Fig. 2a. The jpHMM analysis identified not only the recombination breakpoints but also the recombination intervals, i.e., the intervals around the predicted position where the posterior probability of two assumed subtypes is lower than the threshold value but higher than the probabilities for other viral subtypes [20]. The interval length can be an indirect characteristic of the reliability of the prediction.

**Table 1:** Comparison of recombination site positions in the full genome sequences of Siberian (10.RU.6637) and Uzbek (02.UZ.AY829214) HIV-1 isolates

Genome region^a^	10.RU.6637 genome			02.UZ.AY829214 genome			Homology of region to consensus sequences^b^
	Start of the region, position	End of the region, position	Recombination interval, positions	Start of the region, position	End of the region, position	Recombination interval, positions	
I	790	2208	2204–2209	798	2208	2204–2209	A
II	2209	3108	3098–3186	2209	3233	3199–3269	G
III	3109	4159	4140–4208	3234	4159	4140–4208	A
IV	4160	4451	4446–4509	4160	4861	4846–5526	G
V	4452	6013	5911–6042	4862	5991	5909–6033	A
VI	6014	6193	6194–6216	5992	6193	6194–6216	G
VII	6194	8260	8260–8338	6194	8260	8260–8371	A
VIII	8261	8730	8697–8741	8261	8733	8697–8746	G
IX	8731	9149	9127–9160	8734	9149	9092–9160	A
X	9150	9358	9343–9359	9150	9348	9349–9358	G

^a^All positions are given according to HXB2 (GenBank accession no. K03455)

^b^A, HIV-1 subtype A and G, HIV-1 subtype G

The full genome sequence of HIV-1 can be divided into ten regions according to the recombination breakpoints that were determined (Table 1). As is evident from Table 1, the recombination breakpoints between regions I–II, III–IV, VI–VII, VII–VIII, VIII–IX, and IX–X either match or differ insignificantly, as is the case of region VIII. The recombination intervals for these regions almost completely match and are small in size. The difference between the corresponding positions in regions V and VI is 22 bp, but the recombination intervals almost completely overlap. The situation for pairs II–III and IV–V is rather different (Fig. 2a): not only are the breakpoints in the 10.RU.6637 and 02.UZ.AY829214 genomes mismatched, but the intervals also do not overlap. These results suggest a potential additional recombination between CRF02_AG and HIV-1 subtype A.

In further analysis using SimPlot, the sequence of the HIV-1 10.RU.6637 genome was compared with selected reference sequences. The HIV genome sequences involved in the analysis included not only reference sequences for the main HIV-1 subtypes but also AG and AE recombinant variants. This analysis showed that the HIV-1 10.RU.6637 genome sequence displays a statistically significant similarity to the HIV-1 CRF02_AG sequence as well as to HIV-1 subtype A variants (Fig. 2b).

It is evident from the SimPlot diagram that the major part of the 10.RU.6637 genome sequence is identical to that of HIV-1 CRF02_AG. However, jpHMM analysis suggests that HIV-1 subtype A, according to regions III, V, and IX, is most closely related to the studied Siberian HIV-1 variant. On the other hand, the breakpoints in region IX of 10.RU.6637, the studied HIV-1 Siberian isolate, almost coincide in their positions with the breakpoints identified in the HIV-1 CRF02_AG genome sequence (Fig. 2a).

The recombination breakpoints identified by these two approaches were used for further analysis of individual genome regions. A phylogenetic tree was constructed for each of these regions. Note that the sequences used for constructing these trees did not include the recombination intervals identified by jpHMM. According to these trees, a characteristic of regions I–II, IV, VI–VIII, and X is their clustering in the same branch with the AG subtype. High bootstrap values suggest their close relationship to the AG subtype of Uzbek origin. However, regions III, V, and IX retain a high degree of sequence identity to those of HIV-1 subtype A.

**Fig. 3:**
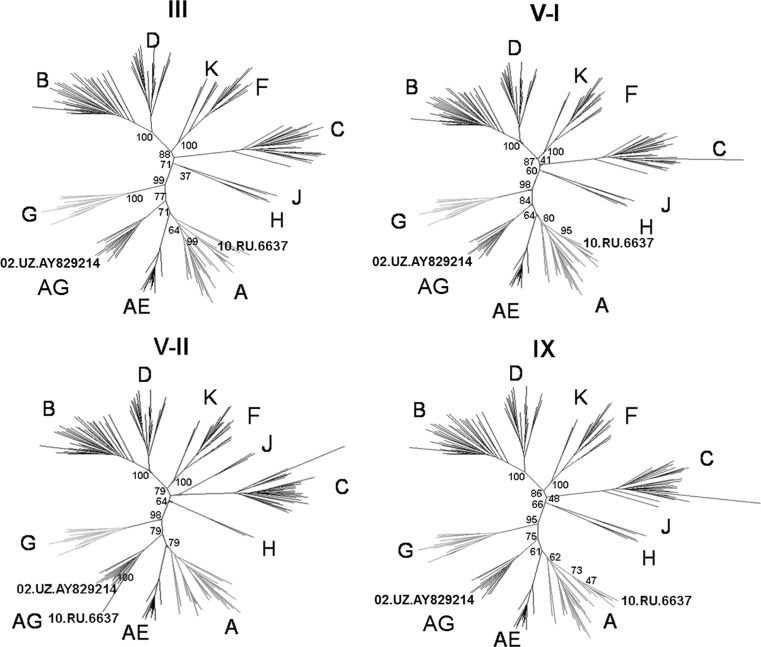
**Phylogenetic trees for the nucleotide sequences of regions III, V-I, V-II, and IX**. These trees were constructed using the maximum-likelihood approach with a bootstrap value of 100. Regions III and IX are similar to those of HIV-1 subtype A. The V-I region of the 10.RU.6637 genome is in the same branch with HIV subtype A variants, whereas the V-II region has retained similarity to that of HIV-1 CRF02_AG

Region V is of particular interest because the 10.RU.6637 and 02.UZ.AY829214 genomes display significant breakpoint mismatches there. The difference between recombination breakpoint positions in this region reaches 410 nt. Region IV in the 10.RU.6637 genome, being similar to the corresponding HIV-1 CRF02_AG sequence, is significantly shorter as compared with that in the 02.UZ.AY829214 genome. According to the profile, region V was divided into two parts, subregions V-I and V-II. According to jpHMM, the former region (positions 4451–5085) of the 10.RU.6637 genome is identical to that of the HIV-1 subtype A, whereas region V-I of the 02.UZ.AY829214 genome is identical to the consensus sequence of HIV-1 subtype G. The latter (subregion V-II), representing the remaining part of region V, displayed identity to the consensus sequence of HIV-1 CRF02_AG in both of the genomes that were compared. Separate phylogenetic trees for regions III, V–I, V–II, and IX using the samples of nucleotide sequences of different HIV-1 genetic variants demonstrated that 10.RU.6637 regions III, V–I, and IX cluster with those of HIV-1 subtype A variants, whereas region V–II retains identity to HIV-1 CRF02_AG sequences (Fig. 3).

## Discussion

With the advance of the HIV infection epidemic, it is becoming ever more evident that geographical distribution of different HIV-1 genetic forms as well as the changes in their composition is a dynamic and unpredictable process. The pattern of an epidemic in a certain area is determined by manifold factors, including the sources of HIV infection, time required for development of infection, economic and geographic features of this area, specific social and behavioral features of the population that determine particular transmission routes, and biological properties of the HIV-1 variants involved.

Several studies have shown that the longer the period during which an epidemic with multiple HIV-1 sources has developed, the higher is the genetic diversity of the circulating viruses [24–26]. On the other hand, a recent epidemic caused by a single HIV source displays a low heterogeneity of spreading HIV variants [27].

Until recently, a genetically homogeneous population of HIV-1 subtype A had been circulating in many regions of Russia [28]. Despite the fact that injection drug users and their sexual partners are still the main driving force for HIV transmission in the majority of Russian regions [29], the overall epidemiological situation in Russia has changed significantly. In particular, while the major part of the drugs in the 1990s came through the western borders of Russia, the current drug traffic goes through Central Asian countries, in particular, Siberia, contributing to an increase in the drug user cohort there [30]. In addition, the mobility of the population has recently increased. Hundreds of thousands of Russian citizens regularly leave the country for other parts of the world, while an increasing number of migrant workers continuously flow from the CIS countries to Russia. Evidently, the number of infection risk factors and the diversity of infection sources are increasing. All these factors cannot but have influenced the epidemiological situation in the Siberian region and have resulted in the emergence and rapid spread of the new HIV-1 recombinant form, CRF02_AG/A. Interestingly, a molecular genetic monitoring of the spreading HIV-1 variants in 2009–2010 has shown that about 50 % of the new sexually transmitted HIV infection cases and cases in the risk group of injection drug users have been caused by new HIV-1 02_AG/A variants [16]. Moreover, solitary viruses have been found that do not cluster with other HIV-1 variants (according to the analyzed region in the *pol* gene sequence), which belong to the AG recombinant form. Note that some isolates with recombination breakpoints different from those of CRF02_AG have also been found in Central Asian countries [13, 23].

Summing up, these data suggest that the penetration of HIV-1 CRF02_AG genetic variant into the Central Asian and/or Siberian populations practicing risky behavior, where the cases of superinfection are rather likely, has resulted in the emergence of second-generation recombinants between HIV-1 CRF02_AG and subtype A. This may explain the identification of structurally distinct HIV-1 AG recombinant forms, including different dead-end variants (that have not become widespread), as well as a population of new HIV-1 recombinant forms, including 02_AG.

To characterize the new HIV-1 variant, isolate 10.RU.6637, we developed a sequencing strategy and determined its full genome sequence. Whole-genome sequencing showed that 10.RU.6637 actually has a unique genetic structure. An integrated analysis of this Siberian HIV-1 variant using special-purpose software has detected at least three genome regions displaying a higher degree of identity to HIV-1 subtype A sequences than to the sequences of HIV-1 CRF02_AG variants circulating in African countries or recorded in Central Asia. This suggests that we encountered a repeated recombination between HIV-1 CRF02_AG and subtype A, which has led to emergence of a new HIV-1, an AG/A recombinant from. Note that this new Siberian variant of CRF02_AG is predominant among the HIV-1 AG recombinant forms spreading in Siberia. Presumably, this is associated with recombination events in the region of the *pol* gene (encoding HIV-1 reverse transcriptase) that could influence the replication properties of the virus.

The abundance of the discovered HIV-1 02_AG variant suggests that this recombination event is important from the standpoint of epidemiology, while the growing prevalence of this 02_AG recombinant form emphasizes the relevance of insights into the specific biological properties of this HIV-1 variant that have enabled viruses to spread across different continents and acquire a leading position among the main genetic variants responsible for the pandemic expansion of HIV infection.
